# Beneficial effects of acute high-intensity exercise on electrophysiological indices of attention processes in young adult men

**DOI:** 10.1016/j.bbr.2018.11.024

**Published:** 2019-02-01

**Authors:** Ebba Du Rietz, Alan R. Barker, Giorgia Michelini, Anna-Sophie Rommel, Isabella Vainieri, Philip Asherson, Jonna Kuntsi

**Affiliations:** a King’s College London, Social, Genetic and Developmental Psychiatry Centre, Institute of Psychiatry, Psychology and Neuroscience, De Crespigny Park, SE5 8AF, London, UK; b Icahn School of Medicine at Mount Sinai Department of Psychiatry New York NY USA; c Children’s Health and Exercise Research Centre, University of Exeter, St. Luke’s Campus, Heavitree Road, EX1 2LU, Exeter, UK

**Keywords:** ES, effect size, EEG, electroencephalography, ERP, event-related potential, ADHD, attention-deficit/hyperactivity disorder, CNV, contingent negative variation, MRT, mean reaction time, RTV, reaction time variability, CPT-OX, cued continuous performance task, Acute exercise, EEG, Cognition, Continuous performance test, Flanker task

## Abstract

**Background:**

Emerging research suggests that a single bout of aerobic exercise can improve cognition, brain function and psychological health. Our aim was to examine the effects of high-intensity exercise on cognitive-performance and brain measures of attention, inhibition and performance-monitoring across a test-battery of three cognitive tasks.

**Method:**

Using a randomised cross-over design, 29 young men completed three successive cognitive tasks (Cued Continuous Performance Task [CPT-OX]; Eriksen Flanker Task; four-choice reaction-time task [Fast Task]) with simultaneous electroencephalogram (EEG) recording before and after a 20-min high-intensity cycling exercise and resting control session. Cognitive-performance measures, EEG power and event-related potential measures, were obtained during the tasks. Random-intercept linear models were used to investigate the effects of exercise, compared to rest, on outcomes.

**Results:**

A single bout of exercise significantly (p < 0.05) increased the amplitude of the event-related potential Go P3, but had no effect on the contingent negative variation (CNV), Cue P3 or NoGo P3, during the CPT-OX. Delta power, recorded during the CPT-OX, also significantly increased after exercise, whereas there was no effect on cognitive-performance in this task. Exercise did not influence any cognitive-performance or brain measures in the subsequent Flanker or Fast Tasks.

**Conclusion:**

Acute high-intensity exercise improves brain-indices reflecting executive and sustained attention during task performance (Go P3 and delta activity), in the CPT-OX, but not anticipatory attention (Cue P3 and CNV) or response inhibition (NoGo P3) in young-adult men. Exercise had no effect on cognitive-performance or brain measures in the subsequent Flanker and Fast tasks, which may potentially be explained by the time delay after exercise.

## Highlights

• High-intensity exercise improved brain measures of attention processes.
• Fitness and physical activity level were not related to degree of improvement.
• We found no effects of exercise on subsequent Flanker and Reaction-time tasks.

## 1 Introduction

Emerging evidence suggests that physical exercise can enhance cognition, brain function and psychological health [[1], [2], [3]]. Recent meta-analyses, including studies using varying controlled (e.g. randomised controlled, within-subject) and non-controlled (quasi-experimental, observational) methodologies, indicate that even a single bout of aerobic exercise, such as running or cycling, improves neurocognitive function in both children and adults [[2], [3], [4], [5], [6], [7]].

Positive effects, particularly of acute (short-lived) exercise sessions of 20 min or more in duration, have been reported in experimental studies on a range of cognitive performance measures, with greatest effects following a delay after exercise cessation [3]. These measures include inhibition and interference control (Effect size; ES = 0.25–0.46) [2,3], attention (ES = 0.42) [2], mean reaction time (MRT) (ES = 0.30–1.41) [5,6] and short-term memory (ES = 0.26) [7]. Several studies suggest that effects are largest for executive functioning, such as response inhibition and interference control [3,8,9], however, relatively few studies have investigated effects on measures of attention and attentional lapses [3]. Overall, findings on cognition have been mixed, as some studies fail to replicate the beneficial effect of acute exercise [10,11]. These inconsistent findings might be explained by differences in the experimental paradigms used, such as the fitness level of participants, time lapse after exercise and the tasks and aspect of cognitive functions being studied [3], as these factors may moderate the effects of exercise.

A growing body of research has also incorporated neurophysiological methods, such as electroencephalography (EEG), which provides a direct measurement of brain activity, to better understand the neural processes enhanced by exercise [12,13]. Experimental studies have reported beneficial effects of acute exercise in children and adults on brain activity relating to attentional and arousal processes. These findings include increased alpha [1,[14], [15], [16], [17]] and beta spectral power [14,16,18], mainly in frontal and parietal areas, during resting-state conditions, which are thought to index background processes such as arousal and activation. However, the direction of these effects on alpha and beta EEG power have been mixed and findings have been inconsistent [1,15,[17], [18], [19]]. No effect has been found for slower-wave delta and theta activity during resting-state [14,19]. Discrepancies in study findings are likely due to heterogeneity in study methodologies [17,19,20], but also a lack of a control group in some studies [15,19,21].

Fewer studies have investigated the effect of acute exercise on event-related potentials (ERPs), which are time-locked brain responses to specific events [29]. Investigations of simultaneously recorded cognitive-performance and ERP measures allow for more in depth understanding of the specific cognitive processes influenced by exercise. The most consistent finding has been an exercise-induced increase in P3 amplitude in Flanker and Go/No-go tasks, during target stimulus presentation (Go P3), which reflects attention allocation and execution [13,[22], [23], [24], [25], [26]]. Enhancements in Go P3 amplitude after acute exercise have in some studies been paralleled with improved behavioural performance of faster reaction times [25,27,28], and increased performance accuracy [23,29], while other studies have suggested that the P3 component shows more sensitivity to the effects of exercise than behavioural measures [13,25]. Although most acute exercise research has focused on the Go P3 component, a small number of studies have also reported beneficial effects of exercise on the NoGo P3 (inhibition of a response [24]), the contingent negative variation (CNV; response preparation [20,30]), and the N2 (conflict monitoring [20,23]). The conflict monitoring N2 is related to error-related components such as the ERN and Pe. Since preliminary beneficial effects on the N2 [20,23] have been reported, it may be that exercise has similar effects on error-related ERP components as well. Overall, the literature on the effect of exercise on brain measures of specific cognitive processes is still limited and few studies have explored effects across several cognitive tasks and ERP components in a single testing session.

A few studies have explored if aerobic fitness of participants, often measured as the peak oxygen consumption (VO_2peak_), moderates the beneficial effects of acute exercise on cognitive performance. While some research suggests that individuals with higher fitness levels improve more from acute exercise on a range of cognitive-performance measures [3,[31], [32], [33]], findings from a meta-analysis on the effects of moderately-intense exercise did not confirm a moderating role of fitness on performance measures of executive function [34]. More limited studies have investigated the moderating role of fitness on the effects of exercise on EEG/ERP outcome measures. While one study failed to find moderating effects of fitness on the relationship between acute exercise and alpha event-related desynchronization [32], another study found that only ‘unfit’ individuals showed higher levels of coherence in the alpha band after rest compared to exercise in NoGo task trials, possibly indicating greater allocation of cognitive resources to the task demands [33]. Further research is needed to clarify the role of individual fitness and physical activity level on the beneficial effects of high-intensity acute exercise on cognitive-performance and brain measures.

### 1.1 Aims

We aimed to investigate the effects of a single bout of high-intensity aerobic cycling exercise on a range of performance and EEG measures implicated in attention, inhibition and performance-monitoring, in a population sample of young adult men. The majority of acute exercise studies testing cognitive and EEG processes have examined moderate-intensity exercise, but high-intensity exercise has been found to have the most beneficial effects on cognitive variables after a time delay post-exercise. Using a cross-over trial design, outcome measures were obtained during three successive cognitive conditions, 30 to 64 min after cycling exercise or rest, which have been used to identify impairments in several psychiatric and neurodevelopmental disorders, such as attention-deficit/hyperactivity disorder (ADHD) [[35], [36], [37]]. In light of the mixed findings in the exercise literature across a wide range of different cognitive tasks, our primary aim was to examine the effects of acute exercise across both performance and brain measures during three consecutive tasks, to better understand the specific cognitive and brain processes that improve from acute exercise. Our secondary aim was to investigate if the degree of improvement from exercise on identified performance and brain measures is related to aerobic fitness and physical activity levels of the participants. This may shed light on characteristics of individuals who would especially benefit from exercise interventions.

As previous studies have reported beneficial effects of acute exercise on performance measures of processing speed, accuracy and ERP measures of Go/NoGo P3, CNV and N2, we predicted that exercise would have beneficial effects on these measures. We used a more exploratory approach for the remaining performance and EEG measures, as these variables have not been previously tested in acute exercise paradigms.

## 2 Materials and methods

### 2.1 Participants

**Fig. 1:**
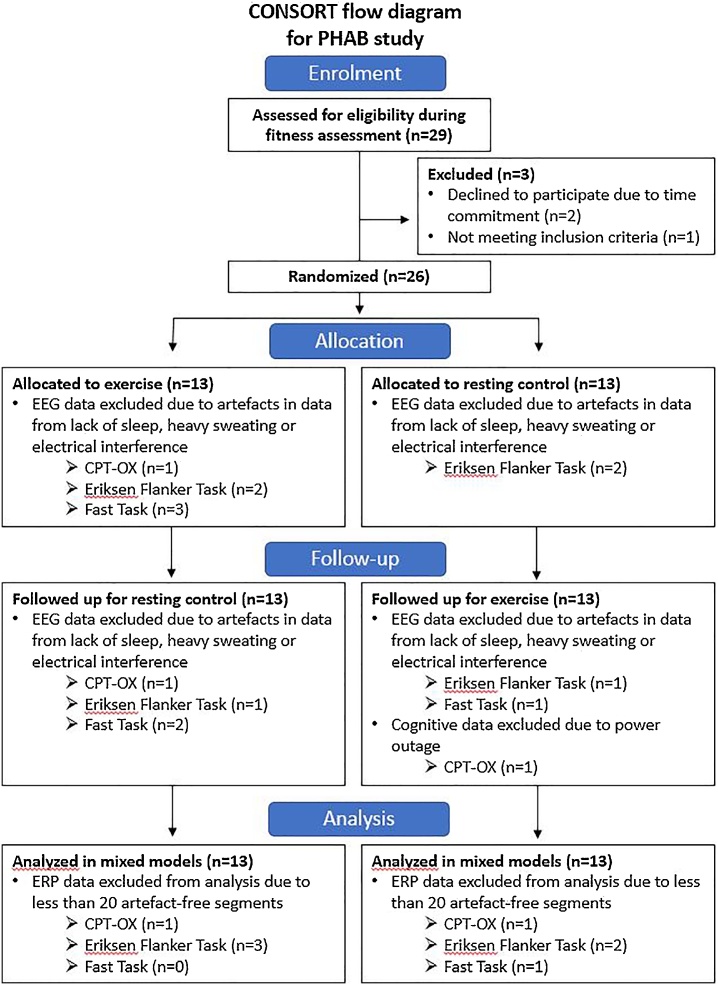
CONSORT flow diagram.

We recruited 29 right-handed men between the ages of 18 and 26 (mean = 21.5; standard deviation [SD] = 2.52) years, of whom 22 were graduate/postgraduate students, 4 were employed and 3 were unemployed. We only included men to reduce sample heterogeneity and increase power in our study. Participants were recruited from a recruitment website for research (callforparticipants.com), through posters in the community near the research centre and through internal advertisement at King’s College London. Participants had an average IQ of 111.72 (SD = 11.33). Exclusion criteria were having any cardiovascular or metabolic disease (e.g. diabetes), being obese (BMI > 30), having bone or joint problems, epilepsy, asthma or any other lung disease. None of the participants were on any psychiatric medication which may have had an influence on cognitive performance. In Fig. 1, we display a CONSORT flow diagram of participants through each stage of the trial for information on participant drop-out (N = 3) and the random allocation of participants to the intervention groups. The study was approved by the Research Ethics Committee at King’s College London (Ref: HR-15/16-3032) and informed consent was obtained from participants before testing.

### 2.2 Procedure

This cross-over trial comprised of three laboratory visits conducted over a range of 11–21 days. The assignment of participants in each trial arm was randomised. Participants attended all three testing sessions at the same time of the day for all three assessments, either in the morning or afternoon.

#### 2.2.1 Visit 1: fitness assessment

At the first initial visit, which occurred 4–14 days (mean = 7.4, SD = 3.6) before the first experimental testing session, each participant underwent an aerobic fitness assessment and health screening procedure. During this first visit, we measured the participant’s stature and body weight to calculate body mass index (BMI = weight(kg)/height(cm)^2^) and asked the participant to complete a health questionnaire (Physical Activity Readiness Questionnaire). Each participant then performed a continuous step-incremental exercise test to exhaustion in order to determine their VO_2peak_ and gas exchange threshold (GET). Participants were asked to cycle on a cycle ergometer (Monark 874E) at a constant cadence of 70 rpm at a starting power output of 70 W for 5 min. Power output was subsequently increased every minute by 28 W until participant exhaustion, which was defined as a drop in cadence below 60 rpm for five consecutive seconds. Test duration was on average 19.3 min (SD = 2.1), including a 5-minute warm-up and 5-minute cool-down period. Measures of VO_2_, carbon dioxide output (VCO_2_), minute ventilation (V_E_) and respiratory exchange ratio (RER) were recorded during the exercise test using a breath-by-breath metabolic cart, facemask and turbine (Cortex Metalyzer 3B, Leipzig, Germany). The participant wore a wireless chest strap to monitor heart rate (Polar, Electro, Finland). Perceived exertion was measured every minute during the cycle test using a 6–20 Borg rating of perceived exertion (RPE) scale [38]. Peak VO_2_ was taken as the highest 10 s average VO_2_ achieved during the test and was used as a measure of aerobic fitness level after normalising for body weight (mL*min^−1^*kg^−1^). The V-slope method was used to determine the GET, which is a non-invasive estimate of the blood lactate threshold [39,40]. The resistance equivalent to 20% delta (Δ; difference between GET and VO_2peak_), which is considered high-intensity exercise [41], was then calculated and verified by two researchers and used for the subsequent exercise trial. The delta concept was used for the exercise condition rather than a fraction of peak VO_2_, as it minimises between participant variation in the physiological response to exercise [41]. High-intensity exercise was chosen because it has been found to have largest effects on cognitive measures when tested after a delay post-exercise [3].

#### 2.2.2 Visits 2 and 3: experimental sessions

During each of the two experimental testing sessions, which were always seven days apart, individuals first performed the three computerized cognitive tasks, while their EEG brain activity was recorded. After the cognitive tasks, participants washed their hair to remove conductive gel, which was then followed by one of two conditions: 1) a 30-min exercise bout consisting of 20-min of exercise at 20% delta with a 5-min warm-up and 5-min cool-down; or 2) a 30-min resting control session. The order of the two testing sessions, i.e. whether a participant attended the exercise or control session first, was counterbalanced. During the exercise intervention, the participant cycled and simultaneously watched a nature documentary (Ocean Giants on BBC, either episodes 2 or 3). During the resting control session the participant was sat on the cycle ergometer without pedalling, while again watching the nature documentary (order of episodes counterbalanced between sessions), in order to standardise paradigms between the two exercise and control sessions. The amount of water consumed by the participant during the first experimental testing session was recorded and they were encouraged to consume the same amount of water on the second experimental testing session. RPE was recorded at the 5^th^, 10^th^, 15^th^ and 20^th^ minute of the exercise and control sessions. Heart rate was measured at the end of the testing session. After both testing sessions, the participant was asked to blow-dry his hair before wearing the EEG cap prior to brain activity measurement. The participant was then asked to perform the same three computerized cognitive tasks performed in the beginning while brain activity was recorded, exactly 30 min post-intervention to allow for the set-up of the EEG cap.

Although participants and researchers were not blinded to the type of intervention that participants were undergoing on the day of testing, researchers were blinded during the pre-processing stage of EEG data.

### 2.3 Measures

*The International Physical Activity Questionnaire* (*IPAQ) short version* was administered during visit 1 to measure the participants physical activity status [42]. IPAQ asks participants about physical activities from the last 7 days. Continuous scores were created to estimate how much time participants spend on (1) vigorous intensity physical activity, (2) moderate intensity physical activity, (3) walking and (4) sitting down. A total score of the full amount of time spent on physical activity was also calculated. IPAQ has acceptable measurement properties of reliability and criterion validity, at least as good as other self-report measures of physical activity [42].

*CPT-OX* [43,44]. This CPT includes rare cued Go and NoGo conditions embedded in a vigilance task with frequent distractors to assess attentional and inhibitory processes. The test consists of 400 letters presented for 150 ms with a stimulus onset asynchrony of 1.65 s in a pseudo-randomised order. The cue letter O occurred with 20% probability (80 Cue stimuli), signaling a subsequent Go/NoGo stimulus, and induced response preparation. Participants were instructed to press a mouse button as fast as possible every time the cue was followed directly by the letter X [(O–X) target sequence, 50% of times after Cue, 40 Go stimuli] but had to withhold responses to O-not-X sequences (NoGo trials, also 50% of times after Cue, 40 NoGo stimuli). Speed and accuracy were emphasized equally. We obtained performance measures of mean reaction time (MRT), reaction time variability (RTV; SD of RT), commission errors (CE) and omission errors (OE). MRT and RTV were calculated across correctly answered Go trials, CE were responses to Cue, NoGo and distractor stimuli or Go stimuli not following a Cue, and OE were non-responses to Go trials. The CPT-OX took approximately 11 min for participants to complete.

*The Eriksen Flanker Task* was an adaptation of the original Eriksen Flanker paradigm designed to increase cognitive load as used in previous studies [45,46]. In each trial, a central black fixation mark was replaced by a target arrow (a black 18-millimeters equilateral triangle). Participants had to indicate whether this arrow pointed toward the left or right by pressing corresponding response buttons with their left or right index fingers. Two flanker arrows identical in shape and size to the target appeared 22 mm above and below the center of the target arrow 100 ms prior to each target arrow. Both flankers pointed in either the same (congruent) or opposite (incongruent) direction to the target. As such, conflict monitoring is maximal during the incongruent condition. When the target appeared, both target and flankers remained on the screen for a further 150 ms, with a new trial being presented every 1650 ms. Two hundred congruent and 200 incongruent trials were arranged in 10 blocks of 40 trials. Speed and accuracy were emphasized equally. Performance measures MRT, RTV and number of errors (left-right errors occurring when participants chose the wrong left or right response) were calculated separately for congruent and incongruent conditions. The Eriksen Flanker Task took approximately 13 min to complete.

*The Fast Task; Baseline Condition* is a slow, unrewarded reaction time task and consists of 72 trials [47], which followed a standard warned four-choice RT paradigm. Four empty circles (warning signals, arranged horizontally) first appeared for 8 s, after which one of them (the target) was colored in. Participants were asked to press the response key that corresponded to the target position. Following a response, the stimuli disappeared and a fixed inter-trial interval of 2.5 s followed. Speed and accuracy were emphasized equally. If participants did not respond within 10 s, the trial terminated. We obtained performance measures of MRT and RTV across correct trials. This version of the Fast Task took approximately 10 min to complete.

### 2.4 EEG

#### 2.4.1 Recording and pre-processing

EEG was recorded from 62 channels DC-coupled recording system (extended 10–20 montage), with a 500 Hz sampling-rate, impedances kept under 10kΩ and FCz as the reference electrode. The electro-oculograms (EOGs) were recorded from electrodes above and below the left eye and at the outer canthi. The EEG data was analyzed using Brain Vision Analyzer (2.0) (Brain Products, Munich, Germany). After down-sampling the data to 256 Hz, the EEG data was re-referenced to the average of all electrodes and filtered offline with digitally band-pass (0.1 to 30 Hz, 24 dB/oct) Butterworth filters. All trials were also visually inspected for electrical artefacts or obvious movement, and sections of data containing artefacts were removed manually by researchers blinded to the type of intervention. Ocular artifacts were corrected using Independent Component Analysis (ICA) [48]. The extracted components were manually inspected and components reflecting ocular artifacts removed by back-projection of all but those components. Sections with other artifacts exceeding ± 100 μV (μV) in any channel were automatically rejected. Channels that had been removed due to excessive artefacts were replaced with topographic spline interpolation after ICA, to estimate virtual EEG activity based on artifact-free activity from other channels.

#### 2.4.2 ERP analyses

ERP analyses involved standard procedures including EEG data segmentation and averaging. Average ERPs were computed separately for each participant, were free from residual artifacts and contained a minimum of 20 artefact-free segments. Please see Table A7 for the average number of artefact-free segments at each of the different time points and conditions, in each task.

##### 2.4.2.1 CPT-OX

**Fig. 2:**
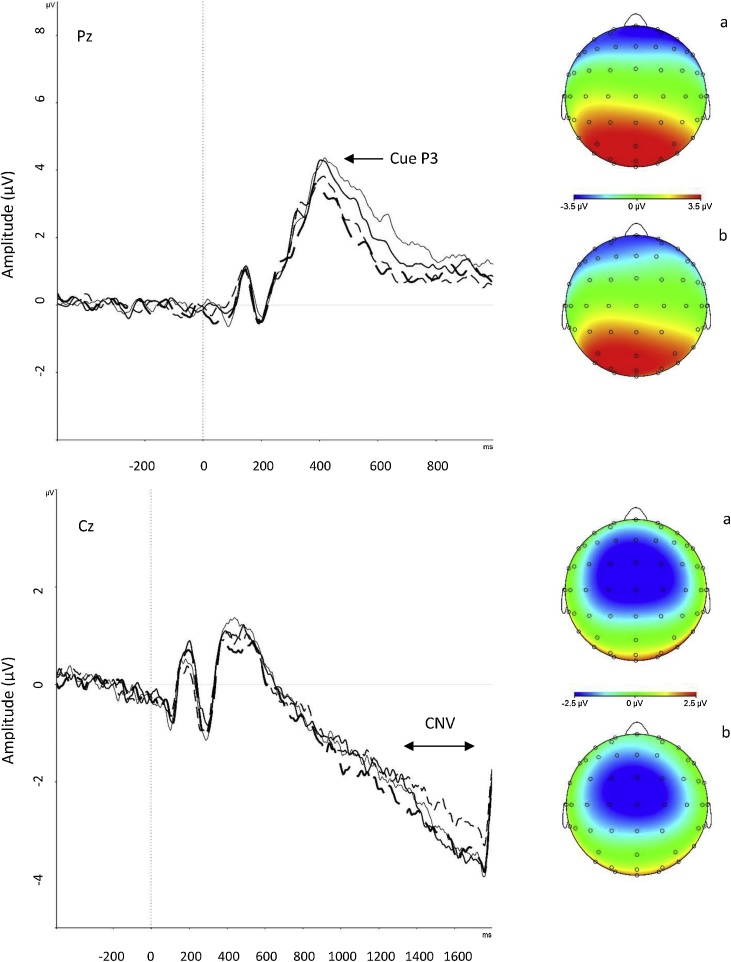
Grand average stimulus-locked event-related potentials of the Cue P3 at the Pz electrode (top half) and contingent negative variation (CNV) at the Cz electrode (bottom half) following cue trials, with topographic maps (a) after exercise intervention and (b) after resting control condition. Thin unbroken line for before exercise intervention, bold unbroken line for after exercise intervention, thin dotted line for before resting control condition and bold dotted line for after resting control condition.

**Fig. 3:**
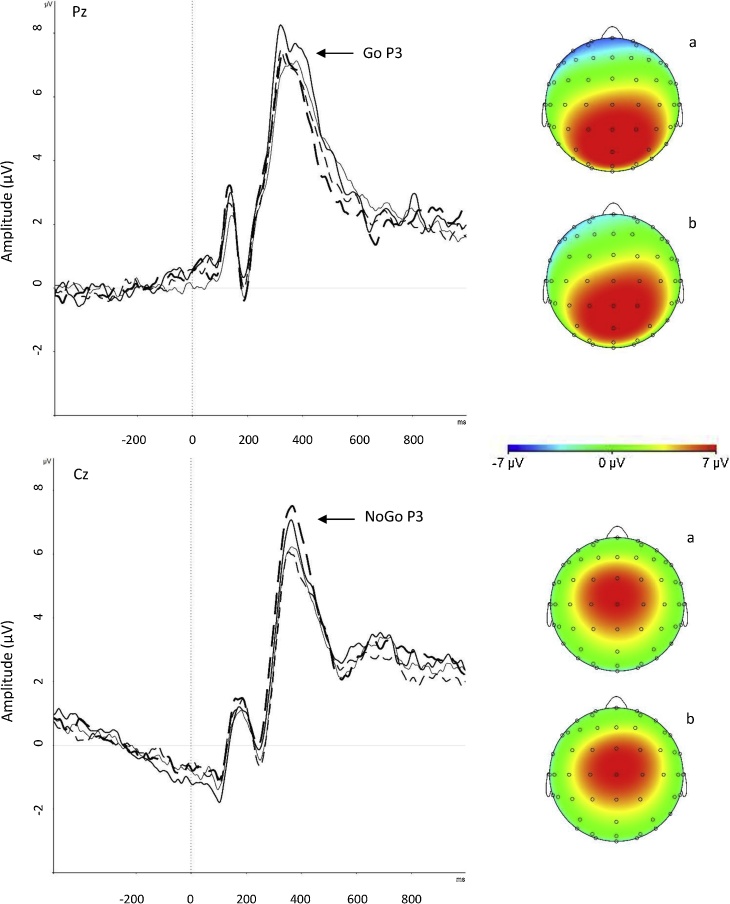
Grand average stimulus-locked event-related potentials of the Go P3 at the Pz electrode after the Go stimulus (top half) and NoGo P3 at the Cz electrode after NoGo trials (bottom half), with topographic maps (a) after exercise intervention and (b) after resting control condition. Thin unbroken line for before exercise intervention, bold unbroken line for after exercise intervention, thin dotted line for before resting control condition and bold dotted line for after resting control condition.

In the CPT-OX, baseline correction was performed using a 500-milliseconds pre-stimulus reference period in line with our previous study using the same task [35]. Stimulus-locked epochs (stimulus window from −200 to 1650 ms) were averaged based on three different response conditions to Cue, Go and NoGo stimuli. Average ERPs only included trials with correct responses or correctly rejected trials. ERP components were identified within the selected electrodes and latency windows for which effects were expected to be largest, based on previous studies [35,[49], [50], [51]], and verified against the topographic maps and grand averages (Fig. 2, Fig. 3). In Cue trials, the P3 was measured as the highest peak amplitude at Pz between 250–600 ms and the CNV was measured as the mean amplitude at Cz between 1300–1650 ms. In NoGo trials, the P3 was measured as the highest peak amplitude at Cz between 250–550 ms. In Go trials, the P3 was measured as the highest peak amplitude at Pz between 250–500 ms.

##### 2.4.2.2 Eriksen Flanker task

In the Flanker task, baseline correction was applied using −300 to −100 ms pre-target (−200 to 0 ms pre-flanker) interval. Analyses of ERN and Pe components were restricted to incongruent trials, as not enough errors are made during congruent trials in this task to allow reliable measurement of ERPs (i.e. at least >20 segments). Data were segmented based on stimulus-locked congruent and incongruent trials where a correct response was made (N2) and response-locked (error-related) incongruent trials where an incorrect response was made (ERN and Pe). The electrodes and latency windows for ERP analyses were selected based on previous studies [36,45,46, topographic maps, and the grand averages. The N2 was measured as maximum negative peak at the FCz electrode between 250–450 milliseconds after target onset. The ERN was measured as a difference from the preceding positivity (PNe, −100 to 50 ms) and measured at FCz between 0 to 150 ms. This peak-to-peak measure was chosen over a maximum peak measure as it has proven to be a robust measure of this component [[52], [53], [54]]. The Pe was measured as maximum positive peak at the CPz electrode between 150 and 450 ms after an erroneous response on incongruent trials.

##### 2.4.2.3 Fast task

In the Fast Task, baseline correction was performed using 200-ms pre-stimulus reference period, in line with other studies using this task [37,55]. P3 amplitude was analysed as the maximum amplitude at Pz between 250 and 500 ms following the target. The electrode and latency window used were selected based on previous work from our group [37,55], topographic maps and the grand averages.

#### 2.4.3 EEG frequency analyses

We estimated absolute EEG power (μV^2^) in each task by computing spectral power in the delta (0.5–3.5 Hertz; Hz), theta (3.5–7.5 Hz), alpha (7.5–12.5 Hz) and beta (12.5–30 Hz) bands. Artefact-free data were segmented into 2-second epochs and power spectra were computed using the Fast Fourier Transform (FFT). In line with previous studies [56,57], absolute EEG power (μV^2^) within each frequency band was averaged across frontal (Fz, F1-F8), central (Cz, C1-C6) and parietal (Pz, P3-P8) regions from individual scalp electrodes to reduce the number of statistical comparisons.

### 2.5 Statistical analyses

We ran 2 × 2 random-intercept linear models in STATA 14 (StataCorp, College Station, TX) to test the effect of acute exercise on cognitive and EEG measures by examining main and interaction effects of Condition (Exercise and Resting control) and Time (Pre- and Post-intervention). For the EEG frequency analyses, we ran 2 × 3 random-intercept linear models to test the main and interaction effects of Condition, Time and Region (Frontal, Central, Parietal areas). If significant Time-by-Condition interaction effects emerged, we ran additional contrasts of marginal linear predictions to compare the change in outcome measures before and after intervention (Pre- minus Post-intervention value) between each Condition. We chose to run mixed models instead of repeated measures ANOVAs because they deal with missing data through maximum likelihood and have less strict assumptions on normality of the data, both of which are advantageous when analysing small samples. The outcome measures CE and OE were severely skewed (+/− 2) [58] and were therefore log-transformed. Cohen’s d effect sizes are presented along with test statistics, to compare group means, where d ≥ 0.20 is a small effect, d ≥ 0.50 is a medium effect and d ≥ 0.80 is a large effect [59].

We further ran Spearman’s rank correlations of each of the fitness level (VO_2peak_) and physical activity (IPAQ) measures with difference-scores (Post- minus Pre-exercise intervention) on outcomes that showed significant improvement after exercise. We controlled for the change in outcome after the resting control condition.

## 3 Results

See Table 1 for descriptive data of the participants and measures obtained during the initial fitness assessment. Table 2 summarises manipulation data obtained during the exercise and resting control sessions.

**Table 1:** Participant characteristics.

Measures	Mean (SD)
Body mass index (BMI), kg/cm^2^	22.54 (2.86)
*Underweight; BMI < 18.5 (n = 2)* * Normal; BMI = 18.5-25 (n = 23)* * Overweight; BMI = 25-30 (n = 4)*	
PA levels, MET-minutes/week	3,480 (2,657)
VO_2peak_, l/min	2.85 (0.69)
VO_2peak_, ml*min^−1^*kg^−1^	39 (8)
GET, l/min	1.70 (0.43)
GET, %VO_2max_	60 (8)
Power output, Watt	262 (56)
HR at end of fitness assessment	183 (13)
RPE at end of fitness assessment	18 (1)

PA levels: Physical activity levels from IPAQ, MET: Metabolic equivalent, VO_2peak_: maximal oxygen uptake, GET: gas exchange threshold, HR: Heart rate; beats/min, RPE: Borg rating of perceived exertion scale.

**Table 2:** Physiological and perceptual outcomes during testing sessions.

Measures	Mean (SD)
***Exercise intervention***	
% Completion of trials	100
RPE at end of session	19 (1)
HR at end of session, beats/min	180 (11)
Mean VO_2_, l/min	1.90 (0.40)
Mean VO_2_, ml*min^−1^*kg^−1^	26 (5)
%VO_2peak_	68 (10)
Peak VO_2_, l/min	2.74 (0.59)
Peak VO_2_, ml*min^−1^*kg^−1^	38 (7)
Power output, Watt	167 (44)
Mean %Δ	21 (26)
***Resting control***	
% Completion of trials	100
Perceived exertion at end of session	6 (1)
HR at end of session	75 (13)
Mean VO_2_, l/min	0.32 (0.06)
Mean VO_2_, ml*min^−1^*kg^−1^	5 (1)
%VO_2peak_	12 (2)

RPE: Borg rating of perceived exertion scale, VO_2peak_: maximal oxygen uptake, GET: gas exchange threshold, HR: Heart rate; beats/min, Δ: difference between GET and VO_2peak_.

### 3.1 CPT-OX

Means and standard deviations of cognitive performance, ERP and EEG frequency measures before and after the exercise and resting control interventions are summarised in Table 3 (see Table A1 for EEG frequency band values within each brain region separately).

**Table 3:** Means and standard deviations of cognitive and brain measures during each Time point (Pre- and Post-intervention) and Condition (Exercise and Resting control) during the CPT-OX.

	Exercise intervention		Resting control	
	Pre	Post	Pre	Post
MRT	370.82 (41.08)	370.56 (45.72)	374.55 (60.12)	378.13 (51.91)
RTV	80.03 (42.54)	105.24 (59.37)	83.73 (51.50)	94.76 (59.12)
OE	1.04 (1.48)	2.04 (3.36)	1.92 (3.58)	3.27 (5.74)
CE	1.04 (1.22)	1.84 (1.93)	1.27 (1.99)	2.27 (2.81)
CNV	−2.80 (1.94)	−3.06 (2.97)	−2.35 (2.23)	−2.89 (2.30)
Cue P3	5.25 (2.64)	5.25 (2.82)	4.99 (2.86)	5.02 (2.92)
NoGo P3	7.43 (4.01)	8.43 (5.86)	7.31 (5.51)	8.65 (5.74)
Go P3	8.18 (4.18)	9.34 (5.49)	8.17 (4.51)	8.45 (4.63)
Delta	3.85 (2.60)	5.44 (4.28)	3.97 (2.22)	4.32 (2.20)
Theta	0.53 (0.50)	0.68 (0.64)	0.63 (0.67)	0.73 (0.70)
Alpha	0.83 (1.61)	1.06 (1.71)	0.94 (1.81)	1.11 (1.82)
Beta	0.11 (0.05)	0.15 (0.09)	0.13 (0.07)	0.14 (0.06)

CPT-OX: Cued Continuous Performance Task, MRT: Mean reaction time, RTV: Reaction time variability, OE: Omission error, CE: Commission error, CNV: Contingent negative variation. Average EEG frequency band measures are reported across all brain regions (see Table S1 for each frontal, parietal and central regional measure).

Two participants had missing EEG data at one visit due to excessive artefacts in the data; one was heavily sweating and the other experienced a lack of sleep. A further participant was excluded from the ERP analysis due to having less than 20 artefact-free segments at both visits. One individual had missing cognitive-performance data during one visit due to technical issues (see Fig. 1).

#### 3.1.1 Cognitive performance data

The random-intercept models did not reveal any significant Condition-by-Time interaction effects on any of the cognitive measures (all p > 0.05): OE, CE, MRT or RTV (Table 4). We found significant main effects of Time for RTV, CEs and OEs, showing that RTV (from M = 81.88 ms [SD = 37.48] to M = 100.88 ms [SD = 49.05]), CEs (from M = 1.15 [SD = 1.64] to M = 2.06 [SD = 2.40]) and OEs (from M = 1.48 [SD = 2.24] to M = 2.72 [SD = 4.08]) increased over time, on average, across the exercise and resting control conditions. No other main effects were observed for cognitive measures (Table 4).

**Table 4:** Main and interaction effects of Time (Pre- and Post-intervention), Condition (Exercise and Resting control) and Region (Frontal, Central and Parietal) on cognitive and brain measures, during the CPT-OX.

		χ^2^	P-value	Effect size
MRT	NS			
RTV	Time	5.19	0.02	0.34
Omission errors	Time	4.12	0.04	0.28
Commission errors	Time	11.30	<.001	0.48
NoGo P3	Time	4.75	0.03	−0.22
Go P3	ConditionxTime	4.97	0.03	a0.24*^b^0.06
CNV	NS			
Delta	ConditionxTime	5.20	0.02	a0.37*^b^0.11
	Time	11.09	<.001	0.26
	Region	66.18	<.001	c0.77*^d^0.26*^e^0.68*
Theta	Condition	4.16	0.04	0.09
	Time	11.83	0.001	0.18
	Region	83.04	<.001	c0.64*^d^0.01 e0.53*
Alpha	Region	52.15	<.001	c0.24*^d^0.26*^e^0.45*
Beta	Time	9.16	0.003	0.24
	Region	60.26	<.001	c0.71*^d^0.19 e0.76*

Presenting significant main and interaction models; p < 0.05. Full table in Table A2.

* p < 0.05 for post-hoc comparisons. Post-hoc comparisons.

a Change after exercise intervention.

b Change after resting control.

c Frontal vs Central.

d Frontal vs Parietal.

e Central vs Parietal, CPT-OX: Cued Continuous Performance Task, MRT: Mean reaction time, RTV: Reaction time variability, OE: Omission error, CE: Commission error, CNV: Contingent negative variation, NS: Not significant.

#### 3.1.2 ERP data

The random-intercept models revealed a significant (p = 0.03) Condition-by-Time effect on the Go P3. Post-hoc comparisons revealed that the Go P3 amplitude significantly increased after exercise (d = 0.24, p = 0.008) but not after the resting control condition (d = 0.06, p = 0.61) (Table 4).

We found no other significant interaction effects on the CNV, Cue P3 or NoGo P3 amplitudes and only one significant main effect of Time on the NoGo P3, showing that the amplitude increased from pre- to post-intervention (from M = 7.40 μV [SD = 4.38] to M = 8.63 μV [SD = 5.65]), across the exercise and resting control conditions (Table 4).

#### 3.1.3 EEG frequency data

The random-intercept models revealed no significant Condition-by-Time-by-Region effects on any of the EEG frequency bands, but did reveal a significant Condition-by-Time interaction effect on delta activity, across regions. Post-hoc comparisons showed that delta activity significantly increased after exercise (d = 0.37, p < 0.001), but not after the resting control session (d = 0.11, p = 0.46) (Table 4). No other interaction effects were significant.

The analyses revealed significant (p < 0.05) main effects of Region across the frequency bands (Table 4). The effect of Time was significant for delta (from M = 3.60 μV^2^ [SD = 1.56] to M = 4.38 μV^2^ [SD = 1.91]), theta (from M = 0.57 μV^2^ [SD = 0.59] to M = 0.70 μV^2^ [SD = 0.68]) and beta (from M = 0.11 μV^2^ [SD = 0.04] to M = 0.14 μV^2^ [SD = 0.06]) activity, showing that EEG activity in these bands increased from pre- to post-time points, across exercise and resting control conditions (Tables 3 & 4). No main effect of Time was found on alpha activity. The effect of Condition was significant only for the theta band, showing that theta activity was higher during the resting control session (M = 0.66 μV^2^ [SD = 0.69]) compared to the exercise session (M = 0.59 μV^2^ [SD = 0.56]), across pre- and post-measures.

To investigate the relationship between the significant increases in delta activity and Go P3 amplitude after exercise, we ran Spearman rank correlations between the change scores (Post- minus Pre-exercise measures). The correlation between change in delta activity and the Go P3 after exercise was not significant (r = 0.15, p > 0.05).

#### 3.1.4 Effect of fitness level and physical activity on improvement from exercise

The Spearman’s rank correlations did not reveal any significant associations between aerobic fitness level and changes in Go P3 amplitude (r = 0.23, p = 0.32) or delta activity (r = 0.21, p = 0.36) after exercise. Physical activity level was not significantly correlated with changes in Go P3 amplitude (r = 0.19, p = 0.43) or delta activity (r = 0.28, p = 0.21) after exercise.

### 3.2 Eriksen flanker task and fast task

Means and standard deviations of cognitive performance, ERP and EEG frequency measures before and after the exercise and resting control interventions are summarised in Tables A3 and A4.

We found no significant Condition-by-Time interaction effects on any of the performance, ERP or EEG frequency band measures in the ERN or Fast Task (Tables A5 & A6).

## 4 Discussion

In this randomised cross-over study of 29 healthy young men, we examined the effect of 20-min of high-intensity cycling exercise, compared to resting, on a range of performance and brain measures, which are either well- or under-studied in the literature, during three consecutive cognitive tasks (CPT-OX; Eriksen Flanker Task; Fast Task). In the CPT-OX, we found that exercise improved executive attention, indexed by enhanced Go P3 amplitude, but not anticipatory attention (Cue P3 and CNV) or inhibitory processing (NoGo P3). Exercise further enhanced delta power during the task, which may suggest improved sustained attention after exercise [[60], [61], [62]]. Neither aerobic fitness nor physical activity levels were significantly correlated with the degree of improvement in the Go P3 amplitude or delta activity measures following exercise. We did not find any effects on performance measures or any of the outcome measures during the later Flanker and Fast tasks. These findings provide insight into the specific processes that may improve following physical aerobic activity.

Our findings suggest that acute high-intensity exercise improves executive attention, indexed by Go P3 amplitude in the Go/NoGo paradigm of the CPT-OX, which relates to increased attention to the task as you respond to the target stimuli. We did not find improvements in ERP components of anticipatory attention or inhibitory processing in the task, contrary to two studies in adults that have found improvements in the CNV during a visuo-spatial attention task [30] and the NoGo P3 during a Go/NoGo task [24]. The discrepancy in findings may be explained by differences in experimental tasks or study design, such as differences in the time lapses between exercise and cognitive task performances. Previous studies had time lapses of 3 [24] and 15–20 [30] min, compared to the longer lapse of 30 min in our study. While most exercise studies have solely focused on the P3 component in isolation from other ERPs, our study, looking across ERP measures, confirms that the enhanced P3 amplitude following exercise is the most robust ERP finding in the exercise literature [13,22], and may possibly have the longest-lasting effect after exercise. The beneficial effects of exercise on executive and sustained attention may be attributed to increased activation of neurotransmitter systems (31,65[31,63]), which in turn induce allocation of resources to attention processes. The reticular-activating hypofrontality (RAH) model [63] of acute exercise specifically proposes a two-step process to explain the psychological effects of exercise. It proposes that exercise first engages arousal mechanisms, involving several neurotransmitter systems, to facilitate implicit information processing. Secondly, exercise disengages higher-order functions of the pre-frontal cortex to keep unhelpful processes from comprising the implicit system’s functioning during simultaneous motor execution. This model, however, refers to mechanisms during exercise execution, rather than after a delay, and may therefore not necessarily be generalisable to our study findings.

We also report for the first time that acute high-intensity exercise enhanced slow delta band activity, across parietal, frontal and central brain regions, during cognitive task performance in the CPT-OX. Drawing on the cognitive-neuroscientific literature, this finding may suggest an improvement in sustained attention after exercise, as increased delta activity during task performance has been linked to attentional processes across experimental paradigms [[60], [61], [62]]. Yet, the improvements in Go P3 amplitude and delta activity were not significantly related, suggesting that these two measures may tap into different aspects of attention. While several previous studies have reported that the P3 component is largely explained by an increased event-related delta response [64,65], less is known about how delta activity throughout a task relates to the event-related P3 components. Previous exercise studies have investigated EEG bands only during resting-state and reported increases in fast-wave brain activity (reflecting wakeful alertness and cortical activation), often immediately after exercise [16,18], but not in slow-wave activity (reflecting drowsiness). We now add to the literature by showing beneficial effects in slow-wave delta activity during cognitive engagement, which instead may reflect attentional processing [[60], [61], [62]]. One implication of our findings is that the identified specific neurophysiological processes that improve from exercise may be modifiable and suitable targets for exercise intervention programs for psychiatric and neurodevelopmental disorders, where these processes are often impaired [66,67].

While we find that neither aerobic fitness nor physical activity level was associated with how well individuals improved in the Go P3 or delta activity measures, previous research on the interacting effect of aerobic fitness on the beneficial effects of exercise on brain measures has been scarce and inconclusive [32,33]. Here we add to the literature by showing that aerobic fitness did not influence the improvement in brain measures of attention, and extend the research by also showing that participants’ average level of physical activity did not influence the effect of exercise. One issue to consider, however, is that we may have been under-powered to detect significant correlations of aerobic fitness and physical activity levels with change in outcome measures after exercise. Based on power calculations (using G*Power 3.1) in our sample of 29 participants, we would only be sufficiently powered to detect moderate-to-large effects. Thus, further research is needed using larger samples to determine the role of aerobic fitness and physical activity level on the effects of acute exercise on brain measures.

The beneficial effects of exercise on brain indices of sustained and executive attention were not reflected by behavioural task performance. While some previous studies have found parallel improvements in processing speed and performance errors with increased Go P3 amplitude [23,25,27], another study has solely found improvements in brain measures, similarly to our current study [15]. Possible explanations for the lack of effects on task performance may be that (1) the CPT-OX task may have been too easy, creating ceiling effects (supported by the fact that 52% of the time individuals made no omission errors during task performance), and (2) brain measures are more sensitive to the effects of exercise in the CPT-OX which might result in too little power to detect the effects on task performance as significant in our relatively small sample.

We found no effects of acute high-intensity exercise on any of the measures during the subsequent Flanker or Fast Tasks following the CPT-OX. One possible explanation for the lack of significant findings in these later tasks is the time lapse between the execution of exercise and the performance of the two tasks, leading to wash out of any potential effects of exercise. Participants performed the Flanker and Fast tasks approximately 41–54 minutes and 54–64 minutes, respectively, after the exercise. This potential interpretation is in line with several previous studies on the effects of acute exercise over an extended period following exercise cessation [68,69]. For example, an EEG study found that activity in the alpha frequency band was inversely correlated with the amount of time elapsed since exercise cessation [70] and a meta-analysis reported that the acute effect of exercise on cognition, across tasks, was only significant within the first 15 min after exercise [3]. Further, in the present study, the increased Go P3 amplitude after exercise in the CPT-OX was not replicated for the target P3 component in the subsequent Fast Task, even though both components reflect attention to target stimuli requiring a response. Another possible explanation for the lack of findings in the Flanker and Fast Tasks may be due to the different nature of the tasks, however, we were unable to investigate this further, as the tasks were not randomly counterbalanced.

One should interpret our findings in light of the study limitations. As we did not counterbalance the order of cognitive tasks, we could not investigate in more detail the influence of time lapse or cognitive load on the effects of exercise, as this was not our primary focus. Further, while we were interested in studying the effects of a 20-minute high-intensity exercise session, we could not separate this effect from that of the total 30-minute exercise including the warm-up and cool-down elements. While this issue is important to note, the warm-up and cool-down elements of the exercise session are standard practice in exercise trials for safety precautions (e.g. avoiding post-exercise syncope). It is also important to consider the generalisability of our sample as only males were included in the study and the average VO_2peak_ was slightly below the average norm values for this age group [71]. It would therefore be informative to investigate the validity of our findings by replicating analyses in females and in a sample with normative levels of fitness for their age group and sex. Lastly, our sample size was relatively small, although similar in size to the majority of acute exercise studies in the literature [14,[16], [17], [18]], thus, larger-scale double-blinded experiments are needed to confirm our findings.

## 5 Conclusion

In this randomised cross-over study, we found that 20 min of acute high-intensity exercise improved brain measures of executive and attentional processes during a continuous performance task, but not measures of anticipatory attention or inhibitory processing. Exercise had no effect on behavioural performance or brain measures in subsequent Flanker and Fast Tasks, which could be due to time delay or level of cognitive load. Insights on the specific processes that improve from exercise may be used to understand the cognitive-electrophysiological targets for intervention programs for psychiatric and neurodevelopmental disorders.

## Declaration of interest

Professor Jonna Kuntsi has given talks at educational events sponsored by Medice; all funds are received by King’s College London and used for studies of ADHD. Professor Asherson has acted in an advisory role for Shire, Janssen-Cilag, Eli-Lilly and Flynn Pharma. He has received education or research grants from Shire, Janssen-Cilag and Eli-Lilly. He has given talks at educational events sponsored by the above companies. Dr Alan Barker, Ebba Du Rietz, Isabella Vainieri, Dr Giorgia Michelini and Dr Anna Rommel report no biomedical financial interests or potential conflicts of interest.
