# Supplementary material for: Beneficial effects of acute high-intensity exercise on electrophysiological indices of attention processes in young adult men
Source: Behav Brain Res. 2019 Feb 1;359:474–84. doi: 10.1016/j.bbr.2018.11.024 (PMC6320386; doi:10.1016/j.bbr.2018.11.024)
Supplement: Supplementary file 1 [file mmc1.docx]

**Appendices**

**Table A1.** Means and standard deviations of EEG frequency bands within each region during each Time points (Pre- and Post-intervention) and Session (Exercise and Resting control) during the CPT-OX.

|  | **Exercise intervention** | | **Resting control** | |
| --- | --- | --- | --- | --- |
|  | **Pre** | **Post** | **Pre** | **Post** |
| Delta |  |  |  |  |
| *frontal* | 5.21 (4.63) | 7.20 (7.63) | 5.35 (3.66) | 5.18 (2.21) |
| *central* | 2.42 (2.25) | 3.31 (2.19) | 2.37 (1.16) | 3.11 (3.24) |
| *parietal* | 3.94 (2.63) | 5.81 (4.23) | 4.18 (2.38) | 4.66 (2.46) |
| Theta |  |  |  |  |
| *frontal* | 0.65 (0.63) | 0.81 (0.74) | 0.73 (0.75) | 0.85 (0.80) |
| *central* | 0.32 (0.23) | 0.42 (0.35) | 0.39 (0.32) | 0.48 (0.39) |
| *parietal* | 0.62 (0.70) | 0.79 (0.92) | 0.76 (0.96) | 0.86 (0.96) |
| Alpha |  |  |  |  |
| *frontal* | 0.79 (1.72) | 0.94 (1.64) | 0.84 (1.70) | 0.98 (1.75) |
| *central* | 0.48 (0.74) | 0.58 (0.70) | 0.55 (0.88) | 0.68 (0.97) |
| *parietal* | 1.21 (2.45) | 1.66 (2.87) | 1.42 (2.88) | 1.67 (2.83) |
| Beta |  |  |  |  |
| *frontal* | 0.14 (0.09) | 0.19 (0.18) | 0.15 (0.11) | 0.16 (0.07) |
| *central* | 0.08 (0.04) | 0.09 (0.05) | 0.09 (0.06) | 0.10 (0.05) |
| *parietal* | 0.12 (0.05) | 0.16 (0.08) | 0.14 (0.07) | 0.15 (0.07) |

EEG: Electroencophalogram, CPT-OX: Cued Continuous Performance Task.

**Table A2.** All main and interaction effects of Time (Pre- and Post-intervention), Condition (Exercise and Resting control) and Region (Frontal, Central and Parietal) on cognitive and brain measures during the Continuous Performance Task.

|  | **Chi^2^** | **P-value** |
| --- | --- | --- |
| ***MRT*** Condition | 1.96 | 0.16 |
| Time | 0.03 | 0.87 |
| ConditionxTime | 0.36 | 0.55 |
| ***RTV*** Condition | 0.14 | 0.71 |
| Time | 5.19 | 0.02 |
| ConditionxTime | 0.73 | 0.39 |
| ***OE*** Condition | 3.24 | 0.07 |
| Time | 4.12 | 0.04 |
| ConditionxTime | 0.02 | 0.89 |
| ***CE***  Condition | 1.08 | 0.30 |
| Time | 11.30 | 0.001 |
| ConditionxTime | 0.14 | 0.71 |
| ***CNV*** Condition | 0.77 | 0.38 |
| Time | 1.63 | 0.20 |
| ConditionxTime | 0.21 | 0.65 |
| ***Cue P3*** Condition | 0.41 | 0.52 |
| Time | 0.15 | 0.70 |
| ConditionxTime | 0.01 | 0.94 |
| ***NoGo P3*** Condition | 0.25 | 0.61 |
| Time | 4.75 | 0.03 |
| ConditionxTime | 0.03 | 0.87 |
| ***Go P3*** Condition | 0.21 | 0.65 |
| Time | 2.28 | 0.13 |
| ConditionxTime | 4.97 | 0.03 |
| ***Delta*** Condition | 3.12 | 0.08 |
| Time | 11.09 | <.001 |
| Region | 66.18 | <.001 |
| ConditionxTime | 5.20 | 0.02 |
| ConditionxRegion | 1.52 | 0.47 |
| TimexRegion | 0.43 | 0.80 |
| ConditionxTimexRegion | 1.98 | 0.37 |
| ***Theta*** Condition | 0.4.16 | 0.04 |
| Time | 11.83 | 0.001 |
| Region | 83.04 | <.001 |
| ConditionxTime | 0.44 | 0.51 |
| ConditionxRegion | 0.28 | 0.87 |
| TimexRegion | 0.33 | 0.85 |
| ConditionxTimexRegion | 0.11 | 0.95 |
| ***Alpha*** Condition | 0.57 | 0.45 |
| Time | 3.59 | 0.06 |
| Region | 52.15 | <.001 |
| ConditionxTime | 0.07 | 0.80 |
| ConditionxRegion | 0.07 | 0.96 |
| TimexRegion | 0.99 | 0.61 |
| ConditionxTimexRegion | 0.22 | 0.89 |
| ***Beta*** Condition | 0.01 | 0.98 |
| Time | 9.16 | 0.003 |
| Region | 60.26 | <.001 |
| ConditionxTime | 1.17 | 0.28 |
| ConditionxRegion | 1.98 | 0.37 |
| TimexRegion | 0.92 | 0.63 |
| ConditionxTimexRegion | 1.59 | 0.45 |

MRT: Mean reaction time, RTV: Reaction time variability, OE: Omission error, CE: Commission error, CNV: Contingent negative variation.

**Table A3**. Means and standard deviations of cognitive and brain measures during each Time point (Pre- and Post-intervention) and Condition (Exercise and Resting control) during the Eriksen Flanker Task.

|  | **Exercise intervention** | | **Resting control** | |
| --- | --- | --- | --- | --- |
|  | **Pre** | **Post** | **Pre** | **Post** |
| Errors (Cong) | 2.84 (2.48) | 3.88 (3.64) | 2.92 (2.45) | 4.04 (3.40) |
| Errors (Incong) | 44.88 (15.66) | 41.92 (15.17) | 42.35 (14.58) | 41.35 (14.27) |
| MRT (Cong) | 321.67 (35.85) | 318.08 (35.11) | 328.37 (39.43) | 322.41 (36.21) |
| MRT (Incong) | 402.38 (41.33) | 394.97 (41.15) | 410.22 (45.69) | 402.36 (36.92) |
| RTV (Cong) | 69.27 (25.11) | 76.20 (32.14) | 74.84 (29.29) | 76.89 (33.20) |
| RTV (Incong) | 62.60 (24.52) | 69.51 (25.62) | 68.37 (25.10) | 70.43 (25.31) |
| N2 (Cong) | -2.49 (1.93) | -1.80 (2.16) | -2.12 (2.21) | -1.77 (1.20) |
| N2 (Incong) | -1.83 (1.87) | -1.39 (1.95) | -1.97 (2.31) | -1.68 (2.06) |
| ERN | -6.22 (3.56) | -5.33 (3.24) | -6.31 (3.27) | -5.83 (2.69) |
| Pe | 7.03 (3.52) | 8.09 (4.61) | 6.45 (4.61) | 7.32 (5.03) |
| Delta | 5.15 (8.12) | 4.57 (3.35) | 5.94 (11.20) | 5.28 (10.94) |
| Theta | 0.67 (0.65) | 0.73 (0.68) | 0.75 (0.85) | 0.76 (0.70) |
| Alpha | 0.89 (1.55) | 1.03 (1.61) | 0.95 (1.81) | 0.98 (1.60) |
| Beta | 0.15 (0.18) | 0.15 (0.14) | 0.13 (0.12) | 0.15 (0.10) |

Cong: Congruent trial, Incong: Incongruent trial, MRT: Mean reaction time, RTV: Reaction time variability. Average EEG frequency band measures are reported across all brain regions.

**Table A4**. Means and standard deviations of cognitive and brain measures during each Time point (Pre- and Post-intervention) and Condition (Exercise and Resting control) during the Fast Task.

|  | **Exercise intervention** | | **Resting control** | |
| --- | --- | --- | --- | --- |
|  | **Pre** | **Post** | **Pre** | **Post** |
| MRT | 49.84 (10.37) | 52.33 (17.16) | 54.70 (22.50) | 55.78 (22.64) |
| RTV | 13.62 (16.20) | 21.70 (31.60) | 24.57 (39.92) | 24.20 (37.99) |
| P3 | 6.30 (3.69) | 4.90 (5.56) | 6.56 (3.90) | 5.69 (4.79) |
| Delta | 3.57 (2.56) | 4.12 (3.49) | 3.42 (2.71) | 4.01 (3.21) |
| Theta | 0.64 (0.73) | 0.66 (0.69) | 0.66 (0.77) | 0.74 (0.86) |
| Alpha | 0.90 (1.66) | 0.89 (1.33) | 0.97 (1.71) | 0.98 (1.64) |
| Beta | 0.13 (0.08) | 0.13 (0.09) | 0.14 (0.09) | 0.15 (0.10) |

MRT: Mean reaction time, RTV: Reaction time variability. Average EEG frequency band measures are reported across all brain regions.

**Table A5.** Main and interaction effects of Time (Pre- and Post-intervention), Condition (Exercise and Resting control) and Region (Frontal, Central and Parietal) on cognitive and brain measures in **Eriksen Flanker Task.**

|  | **Chi2** | **P-value** |
| --- | --- | --- |
| ***Errors (Congruent)*** |  |  |
| Time | 4.87 | 0.03 |
| ***Errors (Incongruent)*** |  |  |
| NS |  |  |
| ***MRT (Congruent)*** |  |  |
| Session | 5.93 | 0.01 |
| ***MRT (Incongruent)*** |  |  |
| Session | 7.16 | 0.01 |
| Time | 6.45 | 0.01 |
| ***RTV (Congruent)*** |  |  |
| *NS* |  |  |
| ***RTV (Incongruent)*** |  |  |
| NS |  |  |
| ***N2 (Congruent)*** |  |  |
| Time | 7.91 | 0.01 |
| ***N2 (Incongruent)*** |  |  |
| Time | 4.35 | 0.04 |
| ***ERN (Incongruent)*** |  |  |
| Time | 6.10 | 0.01 |
| ***Pe (Incongruent)*** |  |  |
| Time | 8.71 | 0.01 |
| ***Delta*** |  |  |
| Region | 22.74 | <.001 |
| ***Theta*** |  |  |
| Region | 88.92 | <.001 |
| ***Alpha*** |  |  |
| Region | 87.76 | <.001 |
| ***Beta*** |  |  |
| Region | 99.90 | <.001 |

MRT: Mean reaction time, RTV: Reaction time variability, NS: Not significant. Only significant main and interaction effects are reported.

**Table A6.** Main and interaction effects of Time (Pre- and Post-intervention), Condition (Exercise and Resting control) and Region (Frontal, Central and Parietal) on cognitive and brain measures in **Fast Task.**

|  | **Chi2** | **P-value** |
| --- | --- | --- |
| ***MRT*** |  |  |
| Condition | 5.69 | 0.02 |
| ***RTV*** |  |  |
| Condition | 3.74 | 0.05 |
| ***P3*** |  |  |
| NS |  |  |
| ***Delta*** |  |  |
| Region | 103.90 | <.001 |
| Time | 5.44 | 0.02 |
| ***Theta*** |  |  |
| Region | 99.85 | <.001 |
| ***Alpha*** |  |  |
| Region | 75.04 | <.001 |
| ***Beta*** |  |  |
| Region | 98.04 | <.001 |
| Condition | 4.95 | 0.03 |

MRT: Mean reaction time, RTV: Reaction time variability, NS: Not significant. Only significant main and interaction effects are reported.

**Table A7**. Means and standard deviations of number of artefact-free segments at each time point (Pre- and Post-intervention) and Condition (Exercise and Resting control)

|  | **Exercise intervention** | | **Resting control** | |
| --- | --- | --- | --- | --- |
|  | **Pre** | **Post** | **Pre** | **Post** |
| ***CPT-OX*** |  |  |  |  |
| Cue P3/CNV | 63.4 (7.41) | 58.64 (11.67) | 64.28 (32.04) | 60.72 (10.07) |
| NoGo P3 | 31.48 (3.96) | 29.71 (5.48) | 32.04 (3.79) | 30.79 (4.06) |
| Go P3 | 31.48 (3.91) | 29.22 (5.48) | 31.80 (4.14) | 29.08 (5.40) |
| ***Flanker Task*** |  |  |  |  |
| N2 (Congruent) | 171.27 (11.18) | 169.58 (13.76) | 169.62 (14.13) | 171.19 (14.88) |
| N2 (Incongruent) | 174.04 (9.99) | 172.96 (14.74) | 174.04 (12.37) | 172.77 (12.70) |
| ERN/Pe (Incongruent) | 39.66 (14.66) | 36.35 (15.31) | 35.96 (17.61) | 37.24 (13.68) |
| ***Fast Task*** |  |  |  |  |
| P3 | 43.12 (4.03) | 43.73 (4.78) | 43.83 (7.68) | 44.19 (6.18) |

CNV: Contingent negative variation.
